# Machine learning for scattering data: strategies, perspectives and applications to surface scattering

**DOI:** 10.1107/S1600576722011566

**Published:** 2023-02-01

**Authors:** Alexander Hinderhofer, Alessandro Greco, Vladimir Starostin, Valentin Munteanu, Linus Pithan, Alexander Gerlach, Frank Schreiber

**Affiliations:** a Institute of Applied Physics, University of Tübingen, Auf der Morgenstelle 10, 72076 Tübingen, Germany; Uppsala University, Sweden; The European Extreme Light Infrastucture, Czech Republic

**Keywords:** surface scattering, X-ray diffraction, neutron scattering, machine learning, data analysis

## Abstract

Machine learning (ML) has received enormous attention in science and beyond. Discussed here are the status, opportunities, challenges and limitations of ML as applied to X-ray and neutron scattering techniques, with an emphasis on surface scattering. Typical strategies are outlined, as well as possible pitfalls. Applications to reflectometry and grazing-incidence scattering are critically discussed. Comment is also given on the availability of training and test data for ML applications, such as neural networks, and a large reflectivity data set is provided as reference data for the community.

## Abstract 2

The status, opportunities, challenges and limitations of machine learning are discussed as applied to X-ray and neutron scattering techniques, with an emphasis on surface scattering.

## 1. Introduction

Machine learning (ML) is receiving enormous attention in essentially all areas of our lives, including in the physical sciences (Erdmann *et al.*, 2021 ▸). The application of ML strategies for the analysis of scattering data is particularly attractive (Chen *et al.*, 2021 ▸). Here we discuss the status, opportunities, challenges and limitations of ML applied to X-ray and neutron scattering techniques, with specific focus on surface scattering (Feidenhans’l, 1989 ▸; Holý *et al.*, 1999 ▸; Birkholz, 2006 ▸; Als-Nielsen & McMorrow, 2011 ▸), which is intended to include interface scattering as well, *i.e.* interfaces between two condensed phases.

One motivation for applying ML in the context of scattering data is simply the hope for faster and more efficient data analysis compared with standard methods. The general theoretical framework for modelling and simulating scattering data is well established. This allows for a simple generation of training data, which is a huge advantage compared with other fields where no direct data generation mechanism exists (*e.g.* computer vision) or where the simulations are very computationally expensive. At the same time, although based essentially on a simple Fourier transform, in addition to optical effects, the conversion of scattering data back into direct information is not straightforward. The acceleration of conventional fitting strategies, which are generally time consuming, with ML methods is also possible if sufficient annotated experimental data are available.

Another motivation is derived from the need to handle huge data volumes and data acquisition rates, which is an almost universal trend in many areas of science. In the scattering world this is due in particular to ever-improving sources with higher flux and to greatly improved detector technology, with area detectors of high resolution and high dynamic range. Real-time experiments (Wang *et al.*, 2021 ▸) and high-throughput experiments (Ludwig, 2019 ▸; Bai *et al.*, 2018 ▸) constitute a particular challenge. In these and many other experiments the rate of data production can be overwhelming and simply impossible to handle for traditional screening by humans, triggering a demand for pre-screening and filtering. A suitable ML algorithm to filter and categorize or even analyse the data before a human researcher inspects the data can be extremely valuable.

There are, of course, many data analysis strategies for different applications in physics. Here, we highlight specific applications of ML techniques, but without a detailed technical discussion of the algorithms, for which we refer the reader to the work of Erdmann *et al.* (2021 ▸). Before discussing ML strategies specifically applied to surface scattering, we mention some other efforts that apply ML to scattering methods.

For example, work on bulk crystallography started many years ago and has showed impressive progress (Tatlier, 2011 ▸; Oviedo *et al.*, 2019 ▸; Lee *et al.*, 2020 ▸; Bai *et al.*, 2018 ▸). Other standard scattering methods, such as small-angle scattering, have also received considerable attention, especially for classification tasks (Song *et al.*, 2020 ▸; Ikemoto *et al.*, 2020 ▸; Franke *et al.*, 2018 ▸; Archibald *et al.*, 2020 ▸; Chang *et al.*, 2020 ▸). For non-standard coherent scattering methods, such as X-ray photon correlation spectroscopy (XPCS), ML-based analysis using autoencoders has been employed (Konstantinova *et al.*, 2021 ▸; Timmermann *et al.*, 2022 ▸). For a more general review of ML methods for scattering we refer to a recent review, which also discusses applications in the broader context of scattering experiments, such as spectroscopy methods, theoretical calculations, automated alignment procedures, beam optimization and data filtering (Chen *et al.*, 2021 ▸).

**Figure 1:**
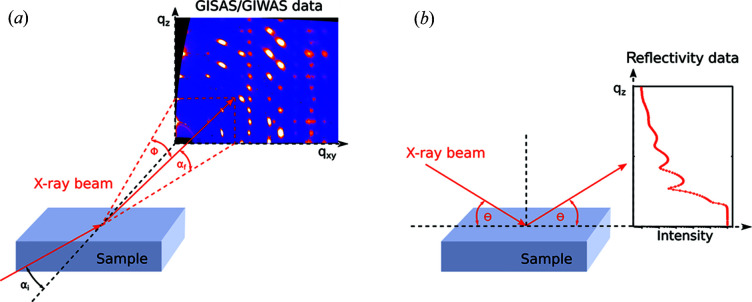
Schematics of typical surface scattering geometries. (*a*) Grazing-incidence small-/wide-angle scattering (GISAS/GIWAS). Data are typically a 2D data set with out-of-plane (*q*_*z*_) and in-plane (*q*_*xy*_) information. (*b*) Reflectometry recorded under the specular condition, *i.e.* the angle of incidence is always equal to the detector angle. X-ray and neutron reflectometry are 1D data sets.

The goal of the present paper is to discuss the perspectives of ML applied to the analysis of scattering data in general and surface scattering data in particular. We first explain the key characteristics and challenges for scattering from surfaces. We then discuss three main surface scattering methods [reflectometry, grazing-incidence small-angle scattering (GISAS) and grazing-incidence wide-angle scattering (GIWAS)], each in their own subsection, with specific regard to ML-based data analysis and their specific scattering geometries (Fig. 1 ▸). In doing so, we implicitly cover both X-rays and neutrons, but we also comment on the specifics of the two different probes (Section 6). This is followed by a critical discussion of the main challenges, as well as the possible role of a reference database and perspectives for establishing it, for which we offer a starting point (Pithan *et al.*, 2022 ▸).

## 2. Strategies and challenges

**Figure 2:**
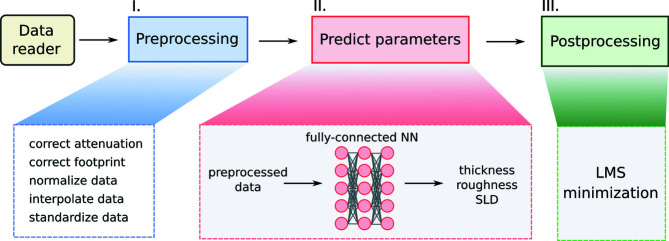
The neural network architecture for extracting thin-film parameters from reflectivity data as demonstrated by Greco *et al.* (2022 ▸).

ML can be applied to solving many different tasks in the context of surface scattering, each with their own specific challenges:

(i) Classification. The ML algorithm can sort the data of an input data set into categories, such as particle shapes in GISAS data, and the output would be a class for each data set.

(ii) Object detection. The ML procedure can find objects in a data set, for example Bragg reflections in grazing-incidence wide-angle X-ray scattering data, and output object coordinates.

(iii) Parameter extraction. The ML algorithm replaces the conventional fitting process and extracts numerical parameters directly from the data. For example, layer thickness and roughness from reflectometry data could be the output of such an approach (Fig. 2 ▸).

(iv) Data processing. For this approach scattering data are typically processed to improve the conventional fitting procedures. For example, the denoising of neutron reflectivity data or XPCS data by an autoencoder has already been demonstrated (Konstantinova *et al.*, 2021 ▸; Timmermann *et al.*, 2022 ▸).

There are several challenges when trying to apply ML to the analysis of scattering data. The most important one is arguably the well known phase problem (Sivia *et al.*, 1991 ▸), which can lead to ambiguous solutions that require additional knowledge for the data to be interpreted correctly.

Furthermore, experimental limitations can reduce the information content in the data. Each setup has specific properties and error sources that need to be taken into account. Differences in the size, shape and divergence of the beam, for example, can lead to slightly different measurement results. Also, different measurements might have a different dynamic range in terms of intensity. This is of course affected by the type of source, but also by optical elements in the beam path, such as slits or monochromators, which may be different between setups. Furthermore, the data may look slightly different for different detectors. Similarly, different sample environments may introduce specific noise or background into the measurement. In addition, before any scattering measurement, each sample typically has to be aligned. While this is considered a routine task, the alignment is usually done iteratively and has a finite accuracy, which may have a surprisingly strong impact (Greco *et al.*, 2022 ▸).

All these factors are difficult to generalize and usually included in the analysis individually for each experimental setup. Thus, if the results of the measurement are sensitive to these effects, it is difficult to train an ML model that is agnostic to the experimental setup. Therefore, to achieve the highest performance, it is usually necessary to include information about the setup in the model.

The above also implies that the training and test data for neural networks must be of high quality. Training data need to be diverse and accurately labelled to be useful. Currently in most work the training is done with simulated data, since large enough annotated sets of real data are not available. On the other hand, for testing the performance of the neural network a much smaller data set is sufficient. We stress here that real experimental data are often very different from simulated data, which makes it an absolute necessity to judge the performance of a neural network on experimental data.

As with basically all ML applications, the dilemma between high generality with poor performance or high specificity with good performance is also found for surface scattering.

By choosing training data and hyper-parameters the boundary for possible outputs is fixed. For example for X-ray or neutron reflectometry, if we train the neural net only with data from layers without interfacial roughness, we cannot expect that model to perform well on rough layers. On the other hand, increasing the flexibility of the neural network by introducing a wider range of training data usually has a very strong negative impact on the performance of the neural network. Finding the optimum between performance and flexibility is therefore a critical task for ML applications.

## 3. X-ray and neutron reflectivity

Specular X-ray and neutron reflectometry (XRR and NR), *i.e.* where α_i_ = α_f_ and ϕ = 0, are common techniques for investigating surfaces, thin films and layered structures (Fig. 1 ▸). The goal of these measurements is typically to extract different physical parameters for each layer in the sample, such as thickness, roughness and scattering length density (SLD), or in the case of neutrons, even magnetic properties. However, depending on the system studied, the data analysis of reflectivity measurements can be difficult and time consuming. For this reason, several attempts have been made to facilitate data analysis using ML in recent years.

**Figure 3:**
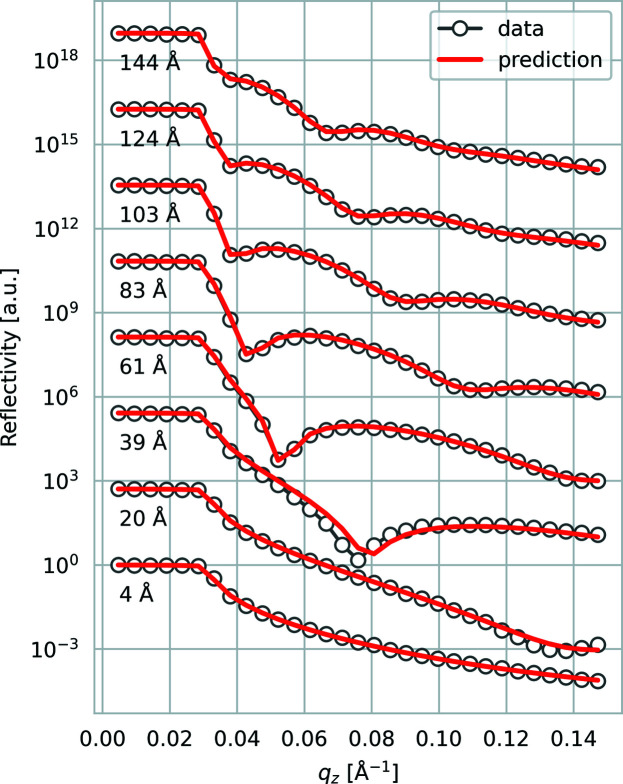
Real-time XRR data sets of a growing organic thin film fitted using the *mlreflect* pipeline (Greco *et al.*, 2022 ▸). The grey circles represent XRR measurements at different times during growth. The numbers in ångströms refer to the corresponding film thickness determined via a manual least-mean-squares fit. The red curves are simulations based on the respective neural network predictions. Figure adapted with permission from Greco *et al.* (2019 ▸) under a Creative Commons Attribution 4.0 International License, https://creativecommons.org/licenses/by/4.0/.

The majority of the publications on this topic focus on the efficient extraction of layer parameters directly from the measured reflectivity curve. The first such published attempt (Greco *et al.*, 2019 ▸) demonstrated a fully connected neural network trained to predict the thickness, roughness and electron density of organic thin films based on real-time reflectivity measurements during growth (Fig. 3 ▸). The neural network architecture is shown in Fig. 2 ▸. The training was done for a fixed substrate using data simulated via a well established theoretical model, such as the Parratt algorithm (Parratt, 1954 ▸). The advantage of this method is that, if trained properly, the neural network is well adapted to solving the inverse problem for a given subset of samples and can predict the sample parameters within a fraction of a second with high accuracy. The disadvantage is that a new neural network model must be trained (or at least re-trained) for different sample architectures (*e.g.* different substrates). Other studies have demonstrated that this approach also works in principle for multiple layers (shown for up to three), but the possible parameter range still had to be restricted (Doucet *et al.*, 2021 ▸; Mironov *et al.*, 2021 ▸). The reason why it is difficult to train a general ML model that is completely agnostic towards the studied system is that, even without considering measurement errors and a finite *q*_*z*_ range, reflectivity problems do not always have a unique solution because of the phase problem (Sivia *et al.*, 1991 ▸). Including background, noise and roughness can further increase the level of ambiguity, even for a simple system, such as a single layer on a substrate (Greco *et al.*, 2021 ▸). Since the above-mentioned neural network models try to approximate an inverse function that maps a given reflectivity curve to a unique set of sample parameters, the solution space necessarily needs to be restricted in such a way as to achieve a mostly unique mapping.

In another approach (Loaiza & Raza, 2021 ▸), this problem is tackled by identifying different symmetry-based families of SLD profiles that can be uniquely distinguished. The main idea is that, if the SLD family of the studied system is known, the neural network can predict the complete SLD profile of any sample within that family. While promising, this approach has, however, not yet been tested with experimental data where the above-mentioned experimental conditions apply.

Kim & Lee (2021 ▸) demonstrated a different neural network architecture employing a mixture density model that predicts a probability density for the sample parameters in the form of several superimposed multi-modal Gaussians in the solution space. This has the advantage of the network yielding several possible solutions at once, with the height of the Gaussians representing the likelihood of a given solution and the widths of the Gaussians yielding implicit error estimates.

Other groups have tried to employ autoencoder architectures for the analysis of reflectivity data. For example, Andrejevic *et al.* (2021 ▸) trained a variational autoencoder to compress reflectivity curves from polarized neutron reflectometry into an information-dense latent space. They deliberately designed the architecture in such a way that the sample parameters can be retrieved from the latent-space variables. Furthermore, the idea is that further fitting in the latent space is easier than fitting the reflectivity curve in *q* space, because there are fewer local minima in the objective function. A different application of autoencoders was shown by Aoki *et al.* (2021 ▸) where an autoencoder was trained to denoise neutron reflectivity measurements which can then be analysed more easily through conventional means. This can help to reduce the integration time that is necessary to achieve a suitable signal-to-noise ratio during the measurement. This is particularly useful for neutron reflectometry where the flux is typically several orders of magnitude lower than for synchrotron radiation.

While already quite varied, all of the approaches published so far still suffer from the problem of being specific to only a subset of samples. In some cases, the neural networks are even specialized to only a certain combination of materials. This shows the necessity of prior physical knowledge to narrow down the task for the neural network. In all of these examples, this physical knowledge is inserted into the model via the selection of the training data. This means that models must be trained for every subset of problems, which can be non-trivial. Furthermore, after a given model is trained, there is no way to use additional knowledge, *e.g.* from other measurements, to exclude certain solutions. Therefore, in the future it would be interesting to explore neural network architectures that allow the input of knowledge about the studied system during inference time, *i.e.* after the model has already been trained. However, successfully training such a model might be challenging and would arguably require a substantially different neural network architecture from what has been published so far.

Most of the training and testing of neural networks in this context is done with simulated data, since large quantities of varied and labelled experimental data are difficult to obtain. Recent work (Greco *et al.*, 2022 ▸) has shown, however, that the performance of a model on simulated data is not a good estimate for the performance on real data. While most of the published work demonstrates results on at least some experimental data sets, it frequently does not represent a large and varied set of reflectivity curves. Without such a representative data set, however, it is difficult to judge the general applicability of a given method. As a result, future work should strive to test their performance on larger data sets. In addition, it might be useful to collect a large body of data that can be shared among research groups for standardized testing, as is common in other ML communities.

**Figure 4:**
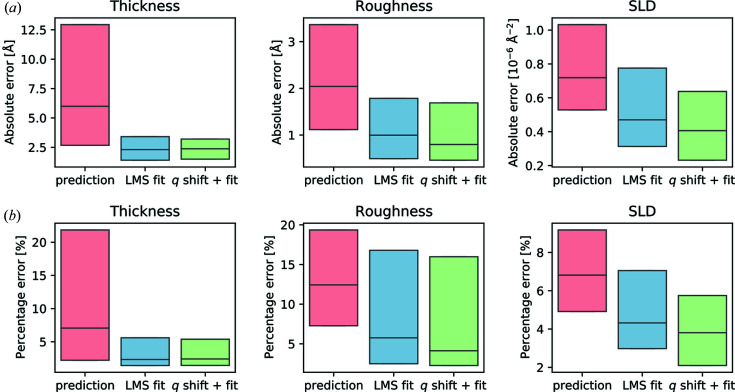
(*a*) Box plots of the absolute errors for 242 measured reflectivity curves for each of the three predicted parameters. The upper and lower edges of the boxes represent the first and third quartiles, respectively, with the horizontal lines inside the boxes denoting the medians. The pink boxes represent the error compared with the pure neural network predictions. The blue boxes represent the error after applying a simple least-mean-squares minimization using the neural network predictions as starting parameters. The green boxes show the error when a *q*_*z*_ shift optimization was performed before this fit. (*b*) The same box plots of the median error but as a percentage of the ground truth. Figure adapted with permission from Greco *et al.* (2022 ▸) under a Creative Commons Attribution 4.0 International License, https://creativecommons.org/licenses/by/4.0/.

As an example for a performance test, Greco *et al.* (2022 ▸) compared a neural network prediction with conventional fitting results from 242 experimental XRR sets (Fig. 4 ▸). The setup there was the fit of three parameters (thickness, roughness, SLD) of a layer on an Si substrate. The median of the initial prediction results (pink boxes in Fig. 4 ▸) is in the range of 7–12% of the ground truth. If the initially predicted parameters are used as starting parameters for a least-mean-squares fit, the median of the error decreases to around 5% compared with the ground truth. This result is acceptable for many applications and can be refined further with postprocessing. For instance the screening for experimental errors in *q* (*q* shift) in combination with a least-squares fit leads to a further decrease in error (green boxes in Fig. 4 ▸).

Other issues for XRR and NR which may be critical for analysis with an ML approach include the limited dynamic range of the measurement, since it essentially defines the maximum *q*_*z*_ range that can be measured. This of course limits the amount of information that can be extracted from the data. In addition, for *in situ* reflectivity measurements, the time it takes to perform a scan can be important. If the observed real-time change in the sample is on the same time scale as the time it takes to measure one reflectivity curve, it may happen that different parts of the curve are measured under different sample conditions. For example, during *in situ* annealing of a sample, changes in roughness or thickness may be continuously ongoing during a reflectivity scan. While a human researcher may notice this effect and apply necessary corrections when analysing the data, it is difficult to include this in an ML model.

## 4. GISAS (GISAXS and GISANS)

Grazing-incidence small-angle X-ray and neutron scattering (GISAXS and GISANS) [Fig. 1 ▸(*a*)] are surface-sensitive techniques used to probe the morphology of surfaces with statistically relevant averaging (Levine *et al.*, 1989 ▸; Sinha *et al.*, 1988 ▸). GISAS has been employed for numerous applications, such as investigating the deposition of metallic nanoparticles on surfaces (Schwartzkopf *et al.*, 2013 ▸) and elucidating the morphology of nanostructured polymer thin films (Müller-Buschbaum, 2003 ▸).

GISAS experiments employ a scattering geometry where the surface sensitivity is achieved by the grazing incidence of the incoming beam and the grazing exit of the outgoing beam. If α_i_ is below the angle of total reflection α_c_ the transmission of the beam is strongly limited and the amplitude from the reflected beam is increased (Tolan, 1999 ▸).

In contrast to bulk methods like powder diffraction, where scattering data in many geometries can often be reduced to a one-dimensional problem, for surface scattering in grazing-incidence geometry such a projection onto one dimension is not possible due to the substrate surface, which breaks the radial symmetry. In addition, the scattering background from the surface is usually anisotropic and can include complex diffuse scattering from the substrate (Sinha *et al.*, 1988 ▸). Therefore, GIWAXS and GISAXS data are 2D, which is associated with specific challenges for ML-based analysis

GISAS data can provide information about the morphological parameters of the surfaces studied, such as the number of layers with different thicknesses and densities, the shape and size distributions of nanoparticles on top of or embedded in the layers, or the densities and spatial ordering of nanoparticles. The conventional analysis obtains the corresponding parameters by solving the inverse problem via iterative adjustments of the parameters and minimizing the difference between the measured and simulated data. This fitting routine is typically slow and would greatly benefit from automated ML-based tools.

**Figure 5:**
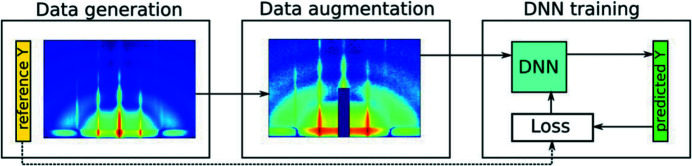
A sketch of the workflow for training a CNN to extract parameters from augmented GISAS data. The GISAS data shown are a simulation of ordered nanoparticles on a surface. Data augmentation is done with several noise sources (including Poisson noise) and cropping of data in the centre to account for the beam stop that is typically present in experimental data. Image reproduced with permission from Van Herck *et al.* (2021 ▸) under a Creative Commons Attribution 4.0 International License, https://creativecommons.org/licenses/by/4.0/.

In general, assumptions about the studied structures might be necessary to reduce complexity and avoid ambiguity in the analysis. Therefore, some of the existing ML solutions for automated GISAS analysis focus on particular morphological models. In this way, convolutional neural networks (CNNs) that extract nanoparticle orientations have been developed (Van Herck *et al.*, 2021 ▸; Liu *et al.*, 2019 ▸). Fig. 5 ▸ shows the typical CNN training workflow with augmented GISAS data (Van Herck *et al.*, 2021 ▸). A possible extension to this approach would involve training an ML model to extract nanoparticle size, interparticle distance and surface layer roughness from GISAS data given the underlying assumptions about the morphological model. Due to unavoidable discrepancies between the simulated and experimental data, further improvement in this direction might require building a database with manually analysed GISAS images, which is a challenging task. An alternative approach involves modern data augmentation techniques that can reproduce experimental artefacts via the generative adversarial network (GAN) technique and its variants (Goodfellow *et al.*, 2014 ▸).

A significantly easier approach is the category classification of GISAS images on the basis of specific characteristics. Ikemoto *et al.* (2020 ▸) used a simple CNN to classify GISAXS data according to the shape of the nanoparticles (capsule, spheroid, ellipsoid, truncated spheroid, hemispheroid, prism, hexagonal prism or cylinder). This approach could be used to select the initial model for an iterative fitting of the GISAXS pattern. Also, a CNN was trained to predict 17 different attributes of X-ray scattering images (including GISAXS measurements) from a predefined list (Wang *et al.*, 2017 ▸). An interactive visualization system for X-ray scattering images with multiple attributes was introduced by Huang *et al.* (2021 ▸). The performance of the multilabel annotation task was improved by Guan *et al.* (2018 ▸, 2020 ▸). The annotation process for the classification tasks is substantially simpler and faster than a comprehensive analysis, and future development in this direction would benefit from aggregating the corresponding data sets into a standard database available for the community.

## 5. GIWAS (GIWAXS and GIWANS)

Grazing-incidence wide-angle X-ray and neutron scattering (GIWAXS and GIWANS) are key methods for investigating crystalline structures on surfaces (Feidenhans’l, 1989 ▸). The scattering geometry is essentially the same as GISAS [Fig. 1 ▸(*a*)] with the only difference being the detected range and resolution in *q*. The wide-angle geometry allows the resolution of Bragg reflections and therefore the analysis of crystal structures and domain orientations. The technique is particularly suitable for *in situ* measurements that enable investigation of crystallization processes or phase transitions in real time, which typically result in hundreds of thousands of images obtained per experimental day.

In contrast to GISAS images with rather complex continuous diffraction features, GIWAS data mostly contain distinct Bragg peaks superimposed on a scattering background and other experimental artefacts such as detector gaps. In general, the characteristics of the Bragg peaks such as their positions, angular and radial sizes, and intensities allow us to obtain information about unit-cell parameters, crystal size distribution or relative fractions of coexisting phases. ML algorithms are ideally suited for processing GIWAXS images by identifying diffraction peaks. However, there are substantially fewer publications on machine learning for GIWAS data than for GISAS. Most of the current approaches focus on preliminary filtering of huge amounts of data. For instance, Wang *et al.* (2017 ▸) used a neural network to classify both GISAXS and GIWAXS images. Other approaches are required for quantitative analysis of GIWAXS data.

The way GIWAXS images are processed depends on the application. In some measurements, the expected structures are known and the task of phase determination is simplified to a comparison of the obtained diffraction peak positions with a predefined list of crystal structures. In other cases, the structures are unknown and a correct structure determination might require indexing algorithms and certain adjustments to the experimental setup (such as larger *q* ranges, absence of coexisting phases *etc.*). Moreover, the diffraction peak characteristics are required for further quantitative analysis. Thus, GIWAS image processing can be split into the peak detection task and further steps using algorithms determined by the specific application. Such an approach allows these complex tasks to be separated into sub-tasks which are easier to improve and test. Separating peak detection and further analysis also allows the use of the same peak detection model for a wide variety of different samples and experimental setups with a wide range of different applications.

**Figure 6:**
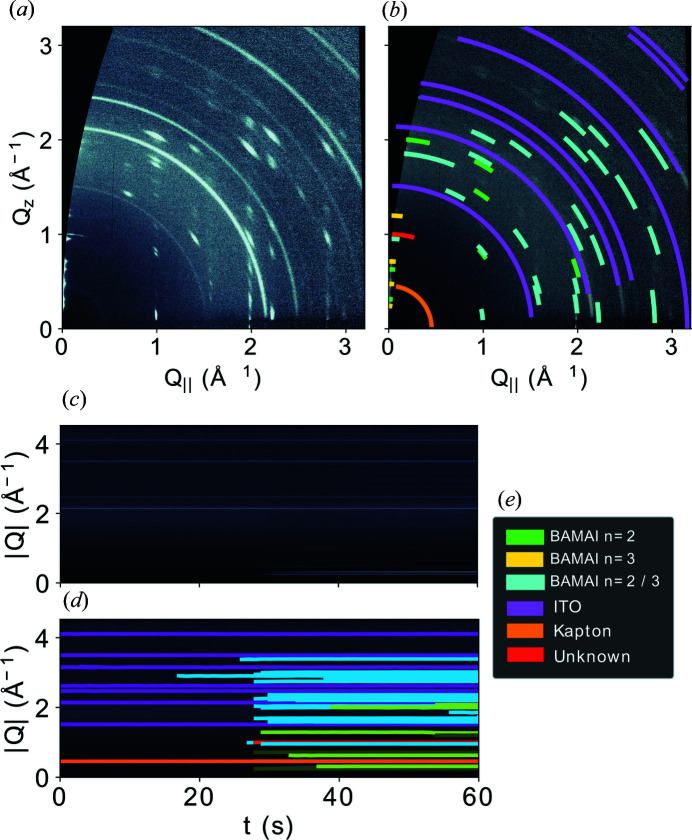
Schematic of GIWAXS peak finding demonstrated for a 2D perovskite thin-film structure during crystallization. (*a*) GIWAXS data, (*b*) annotated with Bragg reflections detected by the ML algorithm at *t* = 60 s. (*c*) The radial positions of the detected peaks over time, with (*d*) the found peak positions. (*e*) Colour code for peak origins. Image reproduced with permission from Starostin *et al.* (2022 ▸) under a Creative Commons Attribution 4.0 International License, https://creativecommons.org/licenses/by/4.0/.

Sullivan *et al.* (2019 ▸) and Liu *et al.* (2020 ▸) employed deep learning methods to accelerate and improve a Bragg peak fitting routine. The first fully automated peak detection approach for GIWAXS images was demonstrated by Starostin *et al.* (2022 ▸). A neural network trained on synthetic GIWAXS images allowed them to obtain a list of detected features (areas with coordinates in a 2D image) which could be passed on to other algorithms for peak indexing, structure matching, unit-cell refinement or texture analysis (Fig. 6 ▸). Similarly to GISAS, the quality of the peak detection analysis can be improved if annotated experimental data are used for the training or at least for formal testing. The use of GANs for data augmentation in this case can be particularly complicated since it is challenging to control the appearance of each diffraction peak on the generated images.

In general, the peak finding procedure is followed by the indexing step, which constitutes a highly challenging task. The existing indexing tools either are based on slow iterative routines (Savikhin *et al.*, 2020 ▸) or require complementary data from the specular geometry (Kainz *et al.*, 2021 ▸), which are unavailable for real-time GIWAXS measurements. Thus, ML techniques might be suitable for accelerating the indexing procedure. Related efforts in this direction are ML-based crystal structure identification methods for 1D X-ray diffraction (XRD) bulk measurements (Tatlier, 2011 ▸; Oviedo *et al.*, 2019 ▸; Lee *et al.*, 2020 ▸). However, to the best of our knowledge, there have been no published attempts to employ ML to accelerate indexing of GIWAS data so far.

The grazing-incidence geometry leads to a highly asymmetric footprint of the beam on the sample surface, and the associated Bragg reflection shape in GIWAXS experiments depends on the resulting resolution, refractive index and penetration depth. The varying shape leads to challenges for the reliable identification of diffraction features in GIWAXS data.

Also, a non-trivial texture, such as a partially preferred domain orientation, may be challenging, in particular in combination with the grazing-incidence geometry and the associated distortion of the scattering signal.

## 6. X-rays versus neutrons

The above appears to be written mostly from the perspective of X-rays but in fact applies largely to both X-ray and neutron scattering, in particular when considering diffraction applications. For inelastic or quasi-elastic scattering (Grimaldo *et al.*, 2019 ▸), or other forms of addressing the dynamics such as XPCS (Sinha *et al.*, 2014 ▸; Timmermann *et al.*, 2022 ▸), there are more significant differences, but these are not the focus of the present paper. Nevertheless, also for diffraction, we should note some specific features of neutron scattering compared with X-ray scattering (Greco *et al.*, 2021 ▸).

In most cases, for both X-rays and neutrons, the scattering follows kinematic theory, except for *e.g.* perfect crystals and optical effects at surfaces (total reflection *etc.*). The key difference concerns the elementary scattering processes and the resulting scattering length and cross sections. These differences lead to the following consequences, all of which can impact the quality of the ML analysis:

(i) For X-rays, the scattering length depends on the number of electrons and can only be positive; for neutrons, the interaction with the nucleus can be both attractive and repulsive. Thus, positive and negative scattering lengths are possible, which can lead to the absence of total external reflection.

(ii) Furthermore, different isotopes of the same element can have very different scattering lengths for neutrons, which allows for contrast tuning through isotopic substitution (Fragneto-Cusani, 2001 ▸).

(iii) For neutrons, absorption is usually smaller than for X-rays.

(iv) Neutron scattering is dependent on the nuclear spin. This can introduce an incoherent part of the nuclear scattering. For diffraction applications this can lead to an enhanced background. We note that for quasi-elastic scattering (energy resolved) this can be exploited to study the dynamics (Grimaldo *et al.*, 2019 ▸).

(v) Because of the magnetic moment of neutrons, the magnetic structure of the sample can be studied (Ankner & Felcher, 1999 ▸). For non-magnetic samples, a magnetic reference layer can be employed (Treece *et al.*, 2019 ▸; Skoda *et al.*, 2022 ▸) which, for a given sample, produces different scattering patterns depending on the polarization of the beam. These patterns can then be co-refined in a common analysis procedure to reduce ambiguities.

(vi) Many neutron sources use pulsed/polychromatic beams with subsequent energy resolution to measure different *q* simultaneously. Since the intensity of each wavelength in the spectrum is not constant, counting statistics can be different for different wavelengths or *q* values (and generally there tends to be a lower incident flux than with X-rays). This affects how the noise is modelled in ML applications (Aoki *et al.*, 2021 ▸) and, interestingly, these ‘sparse sampling’ concepts can also be applied to time-resolved (low-counting) X-ray data (Mareček *et al.*, 2022 ▸).

## 7. Availability of reference data

A major challenge regarding the successful implementation of ML strategies to analyse data arises from the limited availability of suitable test, validation and training data sets consisting of raw experimental data and the corresponding already-performed data analysis. While the training may be aided by simulated (*i.e.* synthetic) data, the true performance test for a neural network to be successful in the analysis of scattering data can only be for real experimental data with their intricacies.

There has been some progress regarding the availability of experimental raw data through data portals and the policies of large-scale facilities (Dimper *et al.*, 2019 ▸) and federated data catalogues like PaNOSC and DataFed (Götz *et al.*, 2020 ▸; Stansberry *et al.*, 2019 ▸) following the FAIR principles (Wilkinson *et al.*, 2016 ▸) and endorsing open science (Bezjak *et al.*, 2018 ▸). In the current state, however, this can be seen rather as a source of data sets from individual experiments (*e.g.* Scoppola *et al.*, 2020 ▸) that provide a significant portion of the metadata generated at large-scale facilities by a specific instrument, but currently do not map the end-to-end process of a scientific experiment (Doucet, 2020 ▸). Even more important is the lack of experiment-specific annotated data to enable ML-based data analysis in surface scattering. Today, if available, individual data sets from a limited number of samples may be found attached to individual scientific publications, *e.g.* Doucet *et al.* (2021 ▸), as there is no common domain-specific data repository, such as the PDB for protein crystallography (Berman *et al.*, 2000 ▸), which allows the retrieval of analysed data and raw data in a systematic fashion (Helliwell *et al.*, 2019 ▸). This lack is also due to the more individual and non-standardized character of the respective experiments in surface scattering compared with *e.g.* protein crystallography.

Nevertheless, larger-scale and comprehensive collections of technique-specific data sets are feasible. For example, *MLExchange* is a platform for easing the use of AI/ML tools by scientific communities by bringing together models, data sets and processing workflows on a single platform (Hexemer *et al.*, 2021 ▸). Other recent initiatives such as DAPHNE4NFDI (DAPHNE4NFDI Consortium, 2023 ▸) or the Tübingen Cluster of Excellence for Machine Learning in Science (Universität T¨ubingen – Cluster of Excellence, 2023 ▸) are also working towards this goal. Scientific community-driven groups such as ORSO (Open Reflectometry Standards Organization; Arnold *et al.*, 2022 ▸) can also help in this context.

As a step towards better availability of reference data, in conjunction with the present publication a collection of ML-ready data sets of X-ray reflectometry are published by Pithan *et al.* (2022 ▸).

## 8. Outlook and summary

We have discussed the status, opportunities, challenges and limitations of ML as applied to X-ray and neutron scattering techniques. In general, ML-based methods are much faster and can be more easily automated compared with conventional fitting and modelling approaches. However the latter are typically more precise, since an expert user controls each step of the analysis. This direct control is, on the other hand, typically time consuming, which makes ML methods preferable for large data volumes and time-critical applications.

In the past few years significant progress has been made in applying ML methods to surface scattering, but some critical milestones are yet to be reached before the scientific community can use ML methods for routine data analysis tasks in surface scattering. Certainly the most relevant missing part is access to large and diverse annotated data sets for testing and comparing the performance of different ML analysis approaches. We are confident that the recent formation of data science consortia will be an important ingredient for the success of ML in scattering.

## 9. Supporting information – details of XRR data set

We have compiled, and published on Zenodo, a collection of experimental XRR curves, together with corresponding box-model parameters that fit the measured data, and these can be used to test, train or validate ML models (Pithan *et al.*, 2022 ▸). From the authors’ point of view the provided data set is intended as a nucleation site for a corresponding reference database. We plan to extend this data set to include a larger variety of models, materials, substrate materials and NR data, and we explicitly welcome external contributions to further versions of the data collection.

## Supplementary Material

A collection of experimental XRR curves together with corresponding box-model parameters that fit the measured data which can be used to train ML models: https://doi.org/10.5281/zenodo.6497437
